# The High-Strain-Rate Impacts Behaviors of Bilayer TC4-(GNPs/TC4) Composites with a Hierarchical Microstructure

**DOI:** 10.3390/ma17225589

**Published:** 2024-11-15

**Authors:** Hongqiang Duan, Xuexia Li, Hongmei Zhang, Xingwang Cheng, Xiaonan Mu, Kefan Zheng

**Affiliations:** 1 School of Materials Science and Engineering, Beijing Institute of Technology, Beijing 100081, China; 2 Tangshan Research Institute, Beijing Institute of Technology, Tangshan 063000, China; 3 National Key Laboratory of Science and Technology on Materials under Shock and Impact, Beijing Institute of Technology, Beijing 100081, China

**Keywords:** titanium matrix composite, microstructure design, mechanical properties, ballistic impact response

## Abstract

Ti matrix composites (TMCs) are promising structural materials that meet the increasing demands for light weight the automobile and aircraft industries. However, the room temperature brittleness in the traditionally homogeneous reinforcement distribution of TMCs limits their application in high-strain-rate impact environments. In the present study, novel bilayer TMCs with hierarchical microstructures were designed by the laminated combination of graphene nanoplatelet (GNPs) reinforced TC4 (Ti-6Al-4V) composites (GNPs/TC4) and a monolithic TC4. Meanwhile, the configuration of the microstructure, impact performance V_50_, and deformation modes of the bilayered TC4-(GNPs/TC4) plate was investigated. The plates were fabricated via field-assisted sintering technology (FAST). It turned out that the TC4-(GNPs/TC4) plate with a 7.5 mm thickness against a 7.62 mm projectile exhibited greater impact performance (V_50_~825 m/s) compared to the TC4 and GNPs/TC4 single-layer plates. The plate failure modes were dependent on the microstructure while the failure behaviors seemed to be influenced by the hierarchical configuration. This work provided a new strategy for utilizing TMCs in the field of high-strain-rate impact environments.

## 1. Introduction

Significant efforts have been directed toward reducing the weight of vehicles, with Ti alloys being advantageous for structural protection due to their lightweight and high specific strength [1,2,3]. For instance, TC4 (Ti-6Al-4V) alloy exhibited high ballistic mass efficiency, and achieved 30~40% weight reductions compared with rolled homogeneous steel [4,5,6,7]. However, the optimization of the microstructure and the property for the traditional TC4 alloy has bottlenecked, which restricts their further application in high-strain-rate impact environments.

Introducing the reinforcement in a TC4 alloy to fabricate Ti matrix composites (TMCs) is a feasible approach to enhance the mechanical properties [8,9,10,11,12,13]. Graphene nanoplatelet (GNPs), as one type of two-dimensional ‘star’ reinforcement, showed great potential for application in the field of TMCs [14,15,16,17,18,19,20] owing to its high fracture strength, high stiffness, and special mechanical properties under extreme dynamic conditions [21]. Our previous work [22] demonstrated that 0.1 wt.% GNPs increased the strength of TMCs by 70% compared to Ti matrix under high-strain-rate conditions (2800~3000 s^−1^), while the dynamic fracture strain was nearly unchanged. this was attributed to the unique two-dimensional honeycomb structure of GNPs, which enhanced the dynamic strength and toughness by suppressing adiabatic shear behavior and hindering crack propagation. Liu et al. [23] found that GNPs/Ti composites owned higher dynamic strength and plasticity compared to the conventional TiB/Ti composites, which was attributed to the fact that the GNPs increased the thermal conductivity and strain-hardening capacity of the materials, and retarded the adiabatic shear band formation. Shen et al. [24] fabricated the 3D network GNPs/TC4 composite by using spark plasma sintering (SPS); they reported that the GNPs greatly influenced the adiabatic shear band susceptibility. Nevertheless, with further additions of GNPs, the improvement in strength inevitably led to a decrease in ductility [25], making it difficult to achieve the balance between high strength and high ductility, which imposed severe limitations on their impact performance.

The configuration design was an effective route to obtain the well-balanced property [26]. In GNPs/Ti composites, inspired by the laminated nacre system offering superior mechanical properties, Zhang et al. [27] fabricated the nacre-like structure GNPs/Ti by using flake powder metallurgy, the quasi-static compressive strength reached to ~2 GPa with high fracture strain ~30%. Mu et al. [28] designed the laminated GNPs/Ti through GNPs spray deposition method in conjunction with a low-temperature consolidation strategy. It was reported that the 2D structure of GNPs were well matched with the laminate architecture, and the strength/ductility of the composite were simultaneously improved compared to pure Ti. Huo et al. [29] reported the ‘brick-and-mortar’ nacre architecture design in the GNPs/Ti composite, which showed the tensile strength 178% higher than that of pure Ti counterpart and maintaining good plasticity. Therefore, it could be found that nacre-like laminated design was able to provide sufficient strength and suitable toughness in GNPs/Ti composites.

Here, a question was proposed: if we could take advantage of the high strength/hardness of laminated TMCs and the high ductility of the Ti-6Al-4V, could we manufacture a TC4-(GNPs/TC4) composite applicable in high-strain-rate environments?

As mentioned above, in the present work, a bilayer TC4-(GNPs/TC4) with a hierarchical microstructure was innovatively designed, and the same matrix material TC4 was chosen for the two neighboring layers to ensure a good bonding on the interface. The nacre-like laminated GNPs/TC4 was applied as a bullet attack face with high strength, hardness, and good toughness, while the rear face was monolithic TC4 (as-rolled commercial TC4), which was highly plastic. This study provided a new case that tried to broaden the application environments of TMCs from traditional high-temperature to high-strain-rate environments and provided new insight into the impact performance of TC4-(GNPs/TC4) composites.

## 2. Experimental Procedure

### 2.1. Processing Routes of the TC4-(GNPs/TC4) Plate

The raw GNPs powders with a thickness of around 3~50 nm and size of 3~10 μm were fabricated by a mechanical exfoliation technique (Tanfeng Tech Co., Ltd., Suzhou, China). The commercially available TC4 powders (average size of ~25 μm) were used as the initial materials and generated by plasma rotating electrode comminuting process atomization. The ductile layer used in this work were commercially available as as-rolled TC4 plates with thicknesses of 4 mm and 7.5 mm. The TC4 plate underwent a pickling process using a combination of hydrofluoric acid (HF), nitric acid (HNO_3_), and water (H_2_O) in a volume ratio of 1:2:7 to eliminate the oxide layer present on the surface. A powder metallurgy technique was used in the fabrication of GNPs/TC4 and TC4-(GNPs/TC4) plates. The manufacturing process included the following stages:

(I) Preparation of GNPs/TC4 powder mixtures

**Figure 1:**
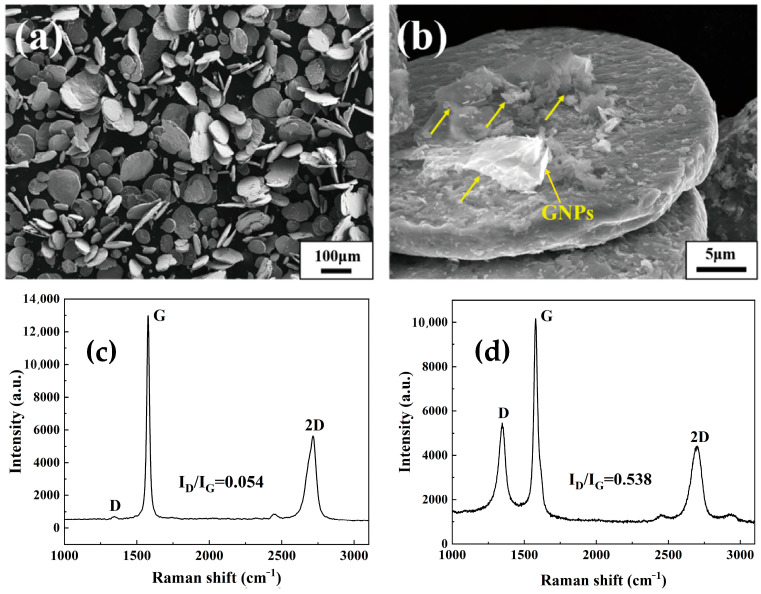
Characterization of the powders: (**a**) SEM image of GNPs/TC4 powder mixtures at low magnification; (**b**) SEM image of GNPs/TC4 powder mixtures at high magnification (GNPs indicated by yellow arrows); (**c**) Raman spectra of the raw GNPs powders; (**d**) Raman spectra of the GNPs/TC4 powders.

The build-in groove ball milling (BGBM) method was applied to fabricate 0.2 wt.% GNPs/TC4 powder mixtures, which could refer to our previous works [30]. Then, the ball milling process was conducted on a planetary ball milling machine (QM-DY4, Nanjing NanDa Instrument Plant, Nanjing, China) at a 350 rpm for 30 min. During the milling process, the originally spherical GNPs/TC4 powder mixtures were rapidly flattened into ‘nacre’ unit flakes using the ‘micro-rolling’ mechanism. Figure 1a shows the morphology of GNPs/TC4 powder mixtures. It was observed that nearly all the spherical powders transformed into flaky powders with a thickness of 1~2 μm and size of 50~100 μm. Additionally, there were no ‘cold-welding’ or internal cracks in these powder mixtures. Figure 1b exhibits the magnified SEM image of the mixed powders. GNPs were attached and/or inserted into the flaky TC4 powder, as indicated by the yellow arrows. Raman spectroscopy is commonly used to assess graphene defects and structures. Figure 1c presents the Raman spectra of raw GNPs powders. The D to G peak intensity ratio (I_D_/I_G_) was estimated as ~0.054, implying the defects of raw GNPs was quite low. As is shown in Figure 1d, while the I_D_/I_G_ value increased to 0.538 after mixing with TC4 powders, indicating that graphene defects increased but the intrinsic structure of the GNPs was still well retained. Furthermore, the chemical composition of the powder mixtures, as well as the monolithic TC4 plate, is shown in Table 1. It can be seen that the O content has only a little enhancement of ~0.15 wt.% after the BGBM, which was attributed to the rapid deformation of powders at a short milling time.

(II) Consolidation of the plates

**Figure 2:**
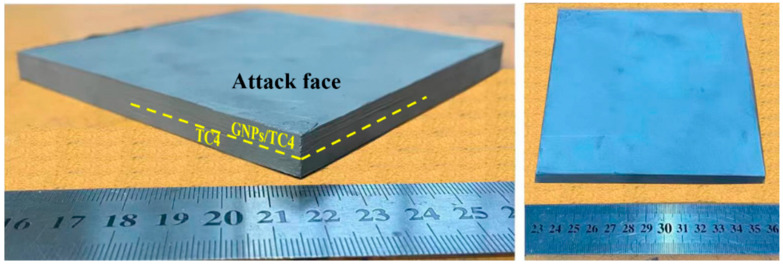
Images of the as-sintered TC4-(GNP/TC4) plate.

For the TC4-(GNPs/TC4) plate, the as-rolled TC4 was firstly machined into 13 mm × 13 mm × 4 mm square plate, then loaded into a graphite die. Then, the as-obtained GNPs/TC4 powders were stacked on the TC4 plate. Details of the powder stacking process have been illustrated in a previous work [30]. Through the subsequent use of Field-Assisted Sintering Technology (FAST, FHP-858, Hateng Tech Co., Ltd., Suzhou, China) utilizing a sintering cycle of 30 min dwell time at 900 °C with a uniaxial pressure of 60 MPa (with no further heat treatment), the nacre-like laminated GNP/TC4 composite with a 3.5 mm thickness was consolidated and firmly bonded on a 4 mm TC4 plate; together, they are referred to as a TC4-(GNPs/TC4) plate with a hierarchical microstructure (shown in Figure 2). Additionally, the TC4 and GNPs/TC4 plate were fabricated using a similar processing route to compare with the TC4-(GNPs/TC4) plate.

### 2.2. Characterization

**Figure 3:**
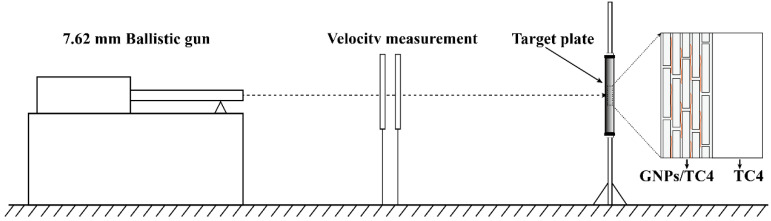
Schematic diagram of the ballistic impact experiment.

The microstructures of the plates were observed using a field emission scanning electron microscope (FESEM, Hitachi S-4800 N, HITACHI, Tokyo, Japan) equipped with an energy dispersive X-ray energy spectrometer (EDS). The interface between GNPs and TC4 was observed by transmission electron microscopy (TEM, Tecnai G20, FEI, Amsterdam, The Netherlands). Raman spectroscopy (Renishaw inVia, excitation laser 514 nm at a laser power of 5.63 mW) (LabRam HR Evolution, Horiba Scientific, Kyoto, Japan) was used to assess the defects and structure of GNPs. Tensile tests were conducted at room temperature using a universal testing machine (Zwick, Roell Z050, Ulm, Germany) at a strain rate of 5 × 10^−4^ s^−1^. The micro-hardness test used a Vickers micro-hardness tester (VMHT30M, LECO Corporation, San Jose, CA, USA) with a load of 300 gf and a dwell time of 15 s. Dynamic compression tests on cylindrical specimens were carried out at room temperature with a strain rate of 3000 s^−1^ using a split Hopkinson press bar (SHPB) (Beijing Institute of Technology, Beijing, China). The cylindrical specimens had dimensions of Φ4 × 4 mm, and the loading direction was parallel to the normal direction (ND). The same structural specimen was tested at least three times to ensure repeatability. For the ballistic impact test, the 7.62 mm standard steel core projectile was used. TC4, GNPs/TC4, and TC4-(GNPs/TC4) were made into target plates to carry out the V_50_ ballistic impact experiments, as is shown in Figure 3. The V_50_ indicated the velocity of the bullets where 50% of the bullets penetrated the target. A pass occurred when the projectile was contained by the target. The failure was considered when damage was caused on the rear face, and the attack faces of the perforated plates were photographed.

## 3. Results and Discussion

### 3.1. Microstructure

**Figure 4:**
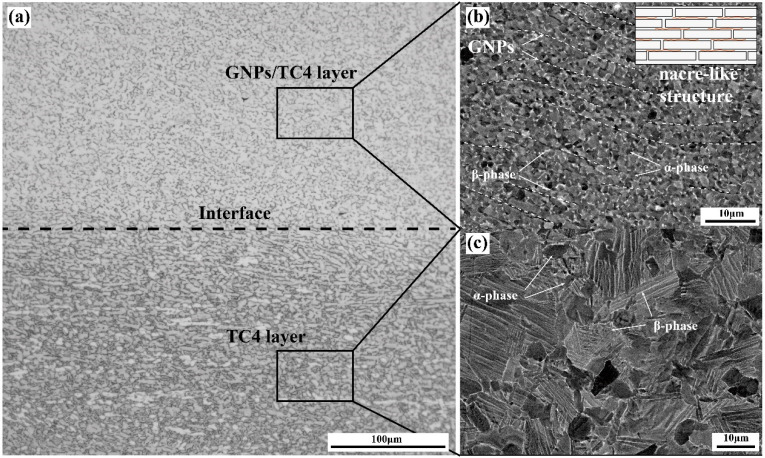
Microstructure analysis: (**a**) OM image of TC4-(GNPs/TC4) composite; (**b**) BSE image of the GNPs/TC4 layer with nacre-like structure; (**c**) BSE image of the TC4 layer.

Figure 4a presents the optical micrographs (OM) of the TC4-(GNPs/TC4) composites. After sintering, metallurgical bonding between the TC4 layer and the GNPs/TC4 layer was successfully achieved. On the interface between the TC4 layer and the GNPs/TC4 layer, no defects, such as voids, cracks, or inclusions, were observed, indicating the excellent interfacial bonding within composite. Backscatter electron (BSE) images of the GNPs/TC4 and TC4 layers are shown in Figure 4b,c. In Figure 4b, the GNPs/TC4 layer exhibits an equiaxial (α + β) dual-phase structure with a significant presence of aligned black bands, representing the existence of GNPs. These GNPs formed a distinctive nacre-like structure within the TC4 matrix (as shown in inset). The TC4 layer, as shown in Figure 4c, displayed a bimodal microstructure, characterized by lamellar α within the β-transformation structure and primary α-phase. The addition of GNPs to the TC4 matrix led to a more refined microstructure compared to the TC4 layer, which was primarily attributed to the effectiveness of GNPs in inhibiting grain boundary migration.

**Figure 5:**
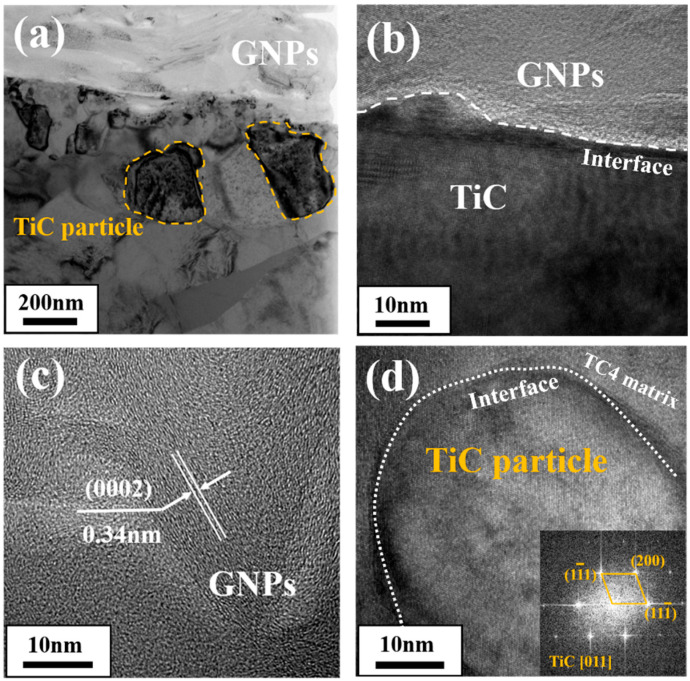
TEM and HRTEM images of the GNPs/TC4 interface: (**a**) right-field TEM image of the GNPs/TiC/TC4 microstructure; (**b**) HRTEM images of interface between GNPs and TiC; (**c**) HRTEM images of the GNPs; (**d**) HRTEM images of the TiC particle.

In order to further investigate the interface between GNPs and the TC4 matrix, TEM characterization was performed, and high-resolution images of the microstructures are shown in Figure 5b–d. The presence of 200 nm fine-grained TiC particles (marked by yellow dashed lines) on the interface between the lamellar GNPs and TC4 are shown in Figure 5a bright field TEM image, which indicated that the GNPs/Ti interface was in a state of chemical bonding. The High-Resolution Transmission Electron Microscopy (HRTEM) image shown in Figure 5b displays that the TiC phase was tightly boned with GNPs, although a 2~3 nm amorphous layer was generated on the GNPs/TiC interface. Figure 5c displays that the lattice spacing of the GNPs (0002) plane was measured as ~0.34 nm, indicating a high crystallinity. GNPs in composite showed continuous and mutually parallel graphene inner-layers. Figure 5d exhibits the HRTEM image of a fine-grained TiC particle (NaCl structure) near the TiC/Ti interface, which was identified by the fast Fourier transformation (FFT) image (taken along [011] zone axis). Note that the TiC particle was well-bonded with TC4 matrix (it was known that TiC and Ti matrix had similar coefficients of thermal expansion (CETs); the CTE of Ti and TiC were 9.0~10.8 × 10^−6^ K^−1^ and 7.4~8.8 × 10^−6^ K^−1^, respectively [31]). Therefore, the well-retained GNPs’ intrinsic structure and firm interface bonding contributed much to the effective load transfer and strength improvement of the composites.

### 3.2. Mechanical Properties

**Figure 6:**
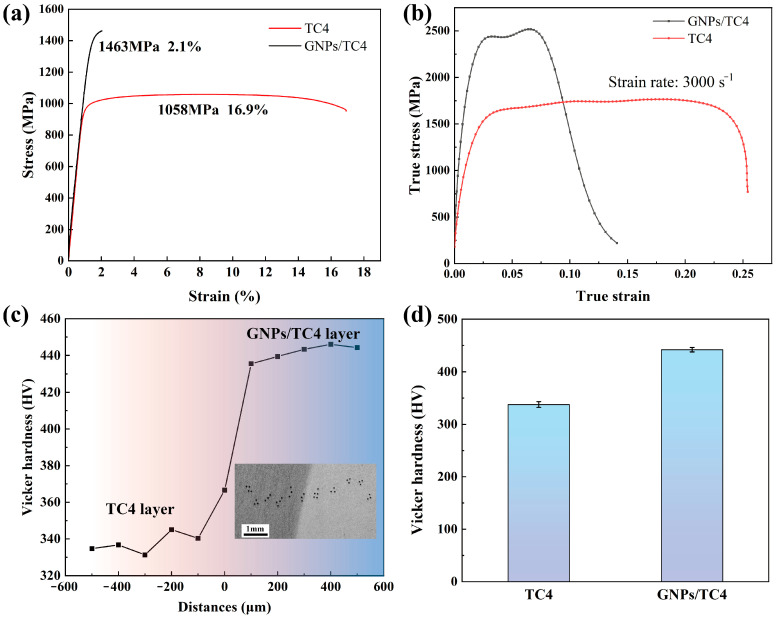
Mechanical properties of the composite: (**a**) tensile stress–strain curves of TC4 and GNPs/TC4 composites; (**b**) true stress–strain curves of dynamic compression at the strain rate of 3000 s^−1^; (**c**) micro-hardness of TC4-(GNPs/TC4) composite varied to the distance from the interface position; (**d**) average hardness values of the TC4 layer and GNPs/TC4 layer.

Figure 6a presents the engineering stress–strain curves from quasi-static tensile experiments for both TC4 and GNPs/TC4 composite layers. The TC4 layer exhibited relatively low tensile strength (1058 MPa) with high elongation (16.9%). In contrast, the GNPs/TC4 composite demonstrated the significantly improved tensile strength but reduced elongation (2.1%). Figure 6b illustrates the dynamic stress–strain curves obtained from the SHPB test, with a strain rate set to approximately 3000 s^−1^. The GNPs/TC4 layer achieved ultra-high dynamic compressive strength (2560 MPa) but a lower critical strain at break (7.8%), while the TC4 layer showed dynamic compressive strength and critical strain values of 1485 MPa and 26.2%, respectively. To further assess the micro-hardness across the TC4-(GNPs/TC4) composite layer interface, microhardness measurements were taken at various distances from the interface in 100 μm increments, as shown in Figure 6c. The hardness increased progressively from the TC4 layer to the GNPs/TC4 layer, stabilizing at a distance of 100 to 200 μm from the interface. The stabilized hardness values for the TC4 and GNPs/TC4 layers were 343 HV and 445 HV, respectively, as is shown in Figure 6d. The gradually increased micro-hardness further implied the good bonding between the TC4 and GNPs/TC4 layer.

### 3.3. Ballistic Impact Response from V_50_ Testing

**Figure 7:**
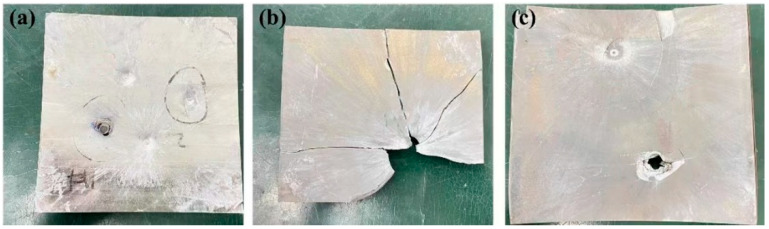
Photographs of the attack face of the impacted targets of TC4, GNPs/TC4, and TC4-(GNPs/TC4) plate: (**a**) TC4 plate; (**b**) GNPs/TC4 plate; (**c**) TC4-(GNPs/TC4) plate.

Ballistics tests were conducted on TC4, GNPs/TC4, and TC4-(GNPs/TC4) plates, and detailed examinations were performed. The post-impact images of the three target plates are depicted in Figure 7a–c. Table 2 summarizes the results from the ballistic trials listing theses three target. Based on 7.5 mm thickness, the V_50_ of theTC4-(GNPs/TC4) plate was 14.7% and 10% better than the TC4 and GNPs/TC4, respectively. The TC4 plate was penetrated at a bullet velocity of approximately 727 m/s, whereas the GNPs/TC4 plate was disintegrated at a velocity of 777 m/s. The observed performance was primarily attributed to the different mechanical properties of the target plates. The low-strength TC4 plate failed to effectively resist bullet penetration, whereas the high-strength GNPs/TC4 plate seemed to have the potential to prevent penetration. However, the lower toughness of the GNPs/TC4 plate (compared to TC4 plate) resulted in rapid fragmentation upon impact, causing the limited protective capability. In particular, the TC4-(GNPs/TC4) plate with bilayer hierarchical microstructure, provided effective protection at 801 m/s and was penetrated at 854 m/s. A comparison of the damage patterns among the three plates revealed that the GNPs/TC4 plate was susceptible to brittleness during impact. However, when the ‘hard’ GNPs/TC4 laminated with the ‘soft’ TC4, the bilayer TC4-(GNPs/TC4) structure showed only limited disintegration at the edges of the impacted surface, and the rear exhibited slight bulging without microcracks. It was suggested that the bilayer hierarchical microstructure effectively dissipated projectile energy, thereby enhancing the protective capabilities of the TC4-(GNPs/TC4) plate.

**Figure 8:**
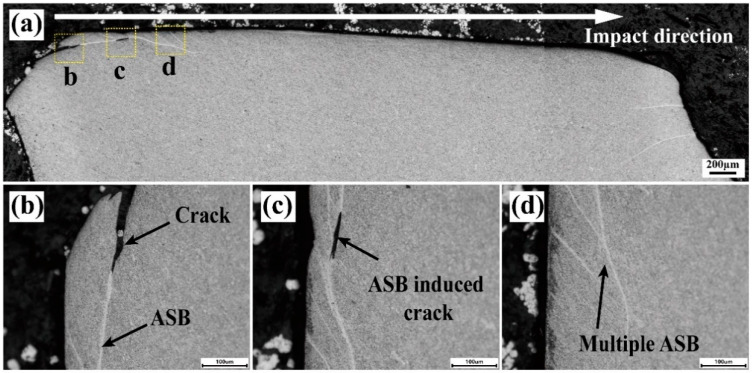
OM graphs showing the microstructure of the impact crater half-section of the TC4 plate: (**a**) cross-sectional images; (**b**–**d**) ASBs and crack morphologies in the vicinity of the impact crater. (The corresponding area were shown in (**a**)).

To comprehensively understand the impact performance of the plates, a detailed microstructural analysis was conducted on the impacted area. The impact crater was sectioned from the center by cutting wires to examine the microstructure of the crater cross-section. Optical micrographs of the TC4 alloy plate depicting the microstructure of half the penetration hole (bullet velocity: 727 m/s) is shown in Figure 8a. Figure 8b,c further illustrate the deformation by numerous adiabatic shear bands (ASBs, shown as white ribbon-shaped features) and ASB-induced cracks distributed along the crater walls, respectively. The observation of multiple ASBs (shown in Figure 8d), including their intersections and bifurcations, suggested that ASBs facilitated the penetration and accelerated the failure of the TC4 target plate. This phenomenon was consistent with findings in other titanium alloys [32,33,34,35,36], particularly under high-strain-rate conditions.

**Figure 9:**
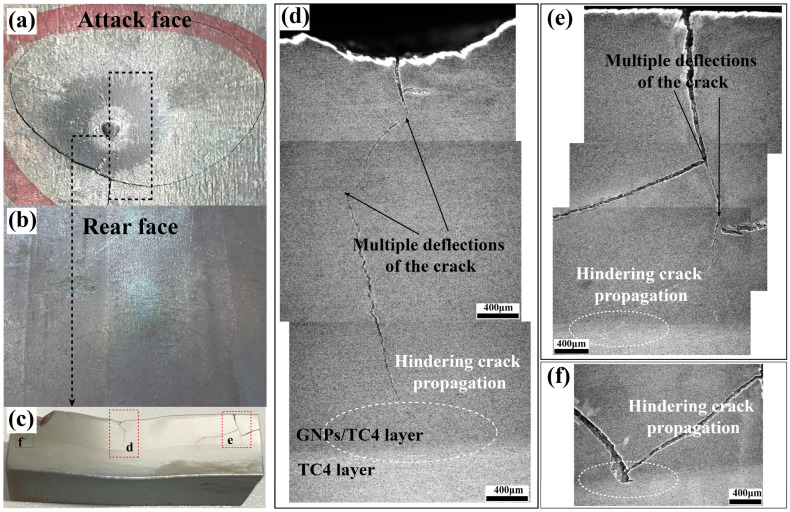
Photographs and SEM images of damage patterns on the attack face and rear face of TC4-(GNPs/TC4) plate after the ballistic testing. (**a**) Attack face (GNPs/TC4 layer) and (**b**) rear face (TC4 layer). (**c**) cross-sectional image; (**d**–**f**) crack propagation morphology in the vicinity of the impact crater. (The corresponding area were shown in (**c**)).

Generally, ASBs were considered the predominant mechanism by which Ti alloys fail and fracture during impact [37,38,39,40,41]. Due to the poor thermal conductivity of Ti alloys, ASBs were easily formed under high strain rates, leading to adiabatic heating that caused softening and strain localization. However, regarding the TC4-(GNPs/TC4) plate, the failure behavior was quite different with the TC4 plate: nearly no ASBs were observed near the micro-cracks. To elucidate the underlying mechanism contributing to the superior impact performance of the TC4-(GNPs/TC4) plate with bilayer structure, OM images of the region near the impact crater were analyzed (bullet velocity 801 m/s), as shown in Figure 9. The TC4-(GNPs/TC4) plate effectively resisted projectile penetration, resulting in the formation of a crater approximately 500 μm deep at the impact site (shown in Figure 9c). A circular crack was observed around the crater (Figure 9a), and a longitudinally extending crack was noted at the center of the entry point of projectile from the cross-sectional view. The rear face of the plate exhibited a slight bulge (Figure 9b) without any evidence of cracking. Comprehensive observations of both the central and surrounding regions indicated that the primary crack at the center of the crater underwent multiple deflections within the GNPs/TC4 layer, ultimately being arrested by the ductile TC4 layer, as depicted in Figure 9d. Additionally, no delamination between GNPs/TC4 and TC4 layer could be detected, suggesting that only the GNPs/TC4 layer crack and TC4 layer plastic deformation were the most important mechanisms allowing for the increased absorption of projectile energy (as shown in Figure 9e,f, which are magnified).

**Figure 10:**
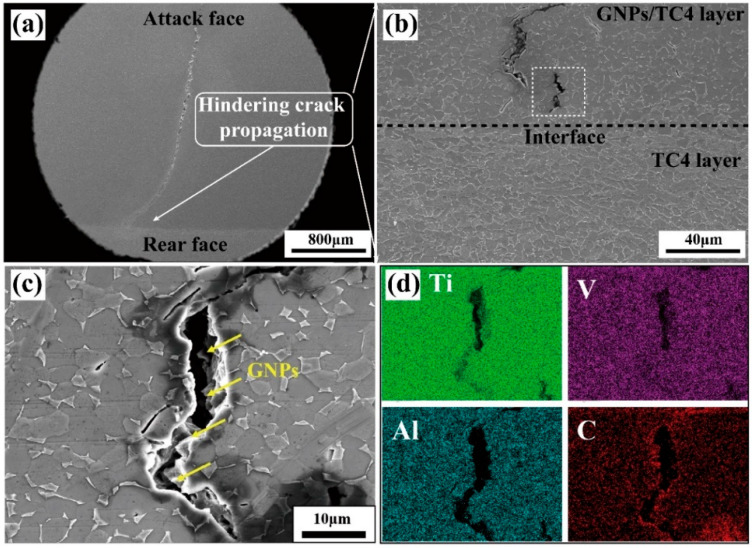
SEM graphs showing the microstructure of the impact crater half-section of the TC4-(GNPs/TC4) plate: (**a**) cross-sectional images; (**b**) cracks propagation in the hard layer (GNPs/TC4) were prevented by the ductile layer (TC4); (**c**) GNPs distribution and (**d**) Ti, V, Al, C elemental distribution near cracks.

Figure 10 presents the SEM images depicting the microstructure of the crater and crack propagation in the TC4-(GNPs/TC4) plate. The primary crack observed in the central region of the crater propagated vertically through the thickness of the plate, ultimately terminating at the TC4 layer (Figure 10a). In the absence of this ductile layer, the main crack would have completely penetrated the target plate, leading to a total failure similar to that observed in the GNPs/TC4 plate (as displayed in Figure 7b). Additional examination of the cracks near the interface revealed a significant presence of microcracks adjacent to the main crack, as illustrated in Figure 10b. Furthermore, fractured GNPs were identified within these microcracks, as shown in SEM image Figure 10c and EDS-mapping Figure 10d. In addition, the absence of ASBs suggested that the incorporation of GNPs influenced the adiabatic shear sensitivity of the TC4 alloy to some extent, thereby preventing adiabatic shear failure under high-strain-rate conditions. According to the previous works, it was believed that GNPs generated thermal-conducting micro-channels within the Ti matrix, thus improving the local thermal conductivity and inhibiting ASBs formation of the composite [22,23]. The TC4-(GNPs/TC4) plate primarily dissipated projectile energy through the main crack and numerous microcracks that were induced by GNPs and the hierarchical microstructure. Meanwhile, the ductile TC4 layer served to impede the propagation of the main cracks, thereby preventing complete penetration through the target plate and ensuring structural integrity.

## 4. Conclusions

In the present work, the high strength/hardness of nacre-like laminated GNPs/TC4s and high ductility of the TC4 were used to fabricate the high impact performance of a bilayer TC4-(GNPs/TC4) plate via a powder metallurgy route. The innovations were summarized as follows:

(1) Design and Fabrication: Novel bilayer TMCs with a hierarchical microstructure were designed by laminating GNPs/TC4 composite with monolithic TC4 alloy by using FAST consolidation method. The 7.51 mm TC4-(GNPs/TC4) plate demonstrated a superior impact performance compared to single-layer TC4 and GNPs/TC4 plates.
(2) Adiabatic Shear Resistance: The GNPs/TC4 layer on the attack face of the TC4-(GNPs/TC4) plate exhibited lower adiabatic shear sensitivity compared to the TC4 layer, and no adiabatic shear failure was observed during the impact. Energy dissipation primarily occurred through the formation of primary cracks and numerous microcracks.
(3) Crack Arresting Mechanism: The highly ductile TC4 layer, positioned as the rear face of the TC4-(GNPs/TC4) plate, effectively arrested the propagation of primary cracks originating from the GNPs/TC4 layer, preventing their failure by penetrating the entire target plate. The strategic lamination of the hard, high-strength GNPs/TC4 layer with a ductile TC4 layer significantly enhances protective capabilities.

## Figures and Tables

**Table 1:** Chemical composition of the TC4 powders, GNPs/TC4 powders, and monolithic TC4 plate.

Sample	Ti (wt.%)	Al (wt.%)	V (wt.%)	C (wt.%)	O (wt.%)	N (wt.%)
Raw TC4 powders	Bal.	6.4	4.0	0.08	0.13	0.03
GNPs/TC4 powders	Bal.	5.9	4.0	0.21	0.28	0.02
Monolithic TC4 plate	Bal.	6.1	3.8	0.08	0.20	0.05

**Table 2:** Summary of the V_50_ ballistic limit results.

Sample (Plate)	Thickness (mm)	Areal Density (g/cm^2^)	Trajectory Distance (m)	V_50_ (m/s)
TC4	7.52	3.38	10	719
GNPs/TC4	7.50	3.29	10	750
TC4-GNPs/TC4	7.51	3.33	10	825

## Data Availability

The original contributions presented in the study are included in the article, further inquiries can be directed to the corresponding author.
